# Escherichia coli ST131 isolates are rare in human meningitis but belong to a distinct O25b-fimH22 clade B lineage shared with avian pathogenic Escherichia coli

**DOI:** 10.1099/mgen.0.001857

**Published:** 2026-10-07

**Authors:** Yasmine Benhadid-Brahmi, Guillaume Geslain, Kevin La, Stéphane Bonacorsi, Céline Courroux, Erick Denamur, Philippe Bidet, André Birgy

**Affiliations:** 1 Université Paris Cité and Université Sorbonne Paris Nord, Inserm, IAME, F-75018 Paris, France; 2 Department of Microbiology, National Reference Center Escherichia coli, Robert Debré University Hospital, Assistance Publique-Hôpitaux de Paris (AP-HP), Paris, France; 3 Pediatric Intensive Care Unit, Robert Debré University Hospital, Assistance Publique-Hôpitaux de Paris (AP-HP), Paris, France

**Keywords:** APEC, *Escherichia coli*, meningitis, newborn, whole-genome sequencing,, ST131

## Abstract

*Escherichia coli* ST131 is a globally disseminated extraintestinal lineage best known for its resistance-driven expansion, yet it is rarely reported as a cause of neonatal meningitis. Whether specific ST131 sublineages retain neuroinvasive potential remains poorly understood. We analysed a 25-year nationwide French collection of 355 neonatal meningitis *E. coli* isolates recovered between 2000 and 2024. ST131 isolates were characterized by whole-genome sequencing, and ST131-O25b-*fim*H22 genomes were contextualized in a phylogeny including human-associated genomes and selected animal- and food-associated references. An extended dataset of 623 animal- and food-associated ST131-O25b-*fim*H22 genomes was analysed separately to assess the distribution of virulence-associated lineages across non-human reservoirs. Nine neonatal meningitis isolates belonged to ST131. Six were O25b:H4-*fim*H22 clade B isolates carrying K1, *invasion of brain endothelium* A (*ibe*A) and pS88-like markers; non-*fim*H22 isolates had heterogeneous virulence/resistance profiles, including two with *sen*B*-cjr*ABC-positive pRS218-like signatures. Phylogenomic analysis placed French ST131-O25b-*fim*H22 meningitis isolates within a conserved 85-genome lineage spanning 1995–2024 and comprising meningitis, bloodstream, urinary and avian-source isolates. This lineage was enriched in K1, *ibe*A, pS88-like signatures and virotype D/D4, with most isolates sharing HC100 assignment (≤100 core-genome allelic differences). The extended animal- and food-associated dataset identified a large poultry-associated HC100 population with the same K1/*ibe*A/pS88-like profile, whereas pRS218-like signatures were uncommon and mainly canine or feline. Although ST131 rarely causes neonatal meningitis, the isolates identified belong to a conserved ancestral ST131-O25b-*fim*H22 clade B lineage combining the major determinants of neonatal meningitis *E. coli* pathogenicity and are closely related to poultry-associated populations. Together with previous observations in ST95, these findings support a shared avian reservoir for highly pathogenic extraintestinal pathogenic *E. coli* lineages, distinct from the resistance-driven expansion of ST131-H30 and highlight the importance of One Health genomic surveillance.

**Impact Statement**

Only a small subset of the globally disseminated *Escherichia coli* ST131 lineage is associated with neonatal meningitis. Here, we show that these isolates belong to a conserved ancestral ST131-O25b-*fim*H22 clade B lineage that combines the major determinants of neonatal meningitis *E. coli* pathogenicity, including the K1 capsular polysaccharide, a hallmark virulence factor enabling serum resistance and blood–brain barrier crossing. This virulence-associated lineage persists despite the worldwide predominance of resistance-driven ST131 lineages. Comparative genomic analyses further reveal that this highly pathogenic, K1-encapsulated lineage is closely related to poultry-associated isolates sharing the same virulence-associated genomic profile. Together with previous observations in ST95, these findings support the existence of avian reservoirs for highly pathogenic extraintestinal pathogenic *E. coli* (ExPEC) lineages and suggest that the evolution of pathogenicity and antimicrobial resistance has followed distinct trajectories within ST131. Our study highlights the importance of integrating human and animal genomic surveillance to better understand the emergence and dissemination of invasive, K1-positive ExPEC clones.

## Data Summary

 Raw Illumina sequencing reads and genome assemblies generated in this study have been deposited in the National Center for Biotechnology Information under BioProject accession number PRJNA1499079. Accession numbers and sources for all publicly available genomes included in the comparative analyses are provided in the Supplementary Material.

## Introduction

*Escherichia coli* is the second leading cause of neonatal meningitis in term neonates and the leading cause in preterm neonates, with an estimated incidence of 3.8 and 26 cases per 100,000 live births, respectively [1].

Most neonatal meningitis *E. coli* (NMEC) strains belong to phylogroup B2 and harbour virulence factors that promote high levels of bacteraemia and blood–brain barrier invasion, including the K1 capsule, invasion-associated genes such as *invasion of brain endothelium A* (*ibe*A), and virulence plasmids such as pS88 and pRS218 [1, 2].

Historically, the antibiotic-susceptible Sequence Type complex (STc) 95, a major lineage within phylogroup B2, has predominated among NMEC worldwide and accounted for a large proportion of neonatal meningitis cases over several decades [3]. In the early 2010s, *E. coli* ST1193 emerged as a novel phylogroup B2 extraintestinal pathogenic *E. coli* (ExPEC) lineage and rapidly achieved global dissemination as a major cause of urinary tract and bloodstream infections, before being increasingly identified in cases of neonatal meningitis [4–6].

In contrast, *E. coli* ST131, another globally disseminated ExPEC lineage, has only rarely been associated with neonatal meningitis despite more than three decades of circulation and extensive evolutionary diversification [3]. Similar to ST1193, ST131 belongs to phylogroup B2 and comprises several major clades (A, B and C) characterized by distinct evolutionary trajectories and pathogenic profiles [7]. The global dissemination of ST131 has been largely driven by fluoroquinolone-resistant, extended-spectrum β-lactamase (ESBL)-producing sublineages, most notably clade C (ST131-O25b-*fim*H30), alongside less resistant clade A (ST131-O16-*fim*H41), both predominantly associated with extraintestinal infections such as urinary tract infections and bacteraemia [8]. In contrast, clade B (ST131-O25b-*fim*H22) is a ST131 sublineage that is typically less drug-resistant but enriched in virulence-associated traits. It is commonly associated with virotype D characterized by the presence of *ibe*A and frequent carriage of the K1 capsule and pS88-like (ColV-type) virulence plasmids [9–11]. Following the conceptual framework proposed by Casadevall and Pirofski [12], we use the term pathogenicity to describe the capacity of a lineage to cause disease, whereas virulence refers to the severity of host damage once infection is established and is typically assessed in experimental infection models.

The pS88 virulence plasmid was originally identified and characterized in the prototype NMEC strain S88 (ST95, O45:K1:H7), where it was shown to play a major role in neonatal meningitis pathogenesis. It harbours a conserved ColV-type virulence backbone encoding iron acquisition systems (siderophores), serum resistance determinants such as *iss*, and additional putative plasmid-associated virulence factors including *hly*F*, omp*Tp*, mig*-14 and the *cva* operon [2, 13]. Similarly, the pRS218 plasmid was identified in the archetypal NMEC strain RS218 (ST95, O18:K1:H7), a human neonatal isolate belonging to a lineage closely related to avian pathogenic *E. coli* (APEC) [14]. This large IncFIB/IIA virulence plasmid encodes virulence-associated determinants involved in iron acquisition, serum survival and extraintestinal adaptation. Functional studies using human brain microvascular endothelial cell (hBMEC) and neonatal rat infection models demonstrated that pRS218 promotes bloodstream persistence, blood–brain barrier invasion and meningitis development [15, 16]. The association of *ibe*A, K1 capsule and pS88-like plasmid in some ST131 sublineages suggests a pathogenicity-associated genetic repertoire compatible with NMEC pathogenicity, although these strains remain rarely reported in this context.

To better understand the epidemiology of ST131 in neonatal meningitis, we analysed ST131 isolates from a 25-year nationwide NMEC collection and placed them within a global phylogenomic framework to characterize their genomic architecture, evolutionary relationships and clinical relevance.

## Methods

### Isolate collection

*E. coli* isolates recovered from neonatal cerebrospinal fluid (CSF) samples in France between 2000 and 2024 were collected as part of the activities of the French National Reference Center (NRC) for *E. coli*, providing a nationwide, multicentric sampling frame. All isolates were stored at −80 °C prior to genomic analysis.

### Whole-genome sequencing and genomic analyses of selected isolates

Genomic DNA libraries from each isolate were prepared using the Nextera DNA Flex Library Preparation Kit (Illumina) and sequenced on an Illumina NextSeq platform. Raw reads were processed with a standardized in-house pipeline, Petanc (https://github.com/iame-researchCenter/Petanc) [17], which performs genome assembly, MLST determination, *fim*H allele, virulence gene detection, antimicrobial resistance gene prediction (including ResFinder), plasmid replicon identification (PlasmidFinder) and core-genome SNP alignment.

A resistance gene score was calculated for each isolate, defined as the total number of acquired antimicrobial resistance genes detected (ranging from 0 to 12), and isolates were further classified according to the presence of ESBL and/or carbapenemase-encoding genes.

This initial analysis enabled comprehensive characterization of all meningitis isolates and identification of those belonging to ST131, which were selected for further analysis.

### Global ST131-O25b-*fim*H22 comparative dataset

To contextualize the neonatal meningitis-associated ST131-O25b-*fim*H22 clade B isolates identified in our collection, we retrieved publicly available genomes belonging to the same lineage from EnteroBase. These included human-associated isolates recovered from urinary tract infections, bacteraemia or meningitis, as well as isolates from animal and food sources.

This dataset was supplemented with previously published ST131-O25b-*fim*H22 genomes from Nhu *et al*. and Liu *et al*. [3, 18]; the latter providing well-characterized poultry- and food-associated references for comparison with meningitis-associated isolates.

All genomes included in the analyses passed sequencing quality-control filters, and assemblies were assessed using standard quality metrics before downstream analyses.

### Phylogenetic analysis and pathogenicity-associated genomic characterization

For each isolate, we assessed the presence of K1 capsule-associated loci (*neu*C), *ibe*A, pS88-like plasmid markers (*hly*F, *omp*Tp, *mig-14*, *iro*N, *iss* and the *cva* operon) and *sen*B*-cjr*ABC-positive pRS218-like plasmid markers using the Petanc workflow (https://github.com/iame-researchCenter/Petanc) [17]. ST131 virotypes were assigned according to the classification schemes of Blanco *et al*. and Mora *et al*. [10, 19] EnteroBase HierCC assignments, including HC5, HC10, HC20, HC50, HC100 and HC200, were retrieved to evaluate genomic relatedness.

A core-genome SNP alignment was generated with Snippy, and a maximum-likelihood phylogeny was reconstructed with IQ-TREE. The phylogeny included the six French neonatal meningitis ST131-O25b-*fim*H22 isolates, human-associated ST131-O25b-*fim*H22 genomes from EnteroBase with available clinical-source metadata, neonatal meningitis isolates reported by Nhu *et al*. [3] and animal- or food-source ST131-O25b-*fim*H22 genomes described by Liu *et al*. [18]. This framework enabled comparison of meningitis-associated isolates with human invasive and urinary isolates, as well as with well-characterized avian- and food-associated lineages.

## Results

### Clinical and epidemiological characteristics of *E. coli* neonatal meningitis cases

Between 2000 and 2024, 355 *E. coli* causing neonatal meningitis were collected through the French NRC for *E. coli*. Among these, nine isolates (2.5%) belonged to ST131, with the earliest isolate recovered in 2001 and the most recent in 2024. Newborns had a median age of 7 days (range, 2–21 days), and only one case occurred within 72 h of life, consistent with predominantly late-onset disease. Five infants were born preterm (<37 weeks), and the male-to-female ratio (7:2) was 3.5. The overall mortality rate was 44.4% (4/9), with all fatal infections occurring in preterm infants. Clinical and major genomic characteristics are summarized in Table 1.

**Table 1.** Clinical characteristics and virulence-associated features of French *E. coli* ST131 NMEC: a pS88-like ColV plasmid was defined by the presence of the following genes or operons: *hly*F, *omp*Tp, *mig*14, *iro*, *iss* and *cva*. A pRS218-like plasmid was defined by the presence of *sen*B and *cjr*ABC, which are characteristic of the pRS218 virulence plasmid [15, 16]

Clinical and virulence-associated feature	S162	S242	S246	S261	S276	S296	S398	S424	S573
Term (weeks)	34	30	33	38	39	38	38	31	28
Sex	F	M	F	M	M	M	M	M	M
Year	2001	2006	2006	2006	2007	2009	2015	2016	2024
Age (days)	5	6	7	4	14	2	21	17	15
Outcome	death	death	alive	alive	alive	alive	alive	death	death
O:H serotype	O25b:H4	O25b:H4	O25b:H4	O16:H4	O25b:H4	O25b:H4	O25b:H4	O25b:H4	O25b:H4
Capsule	K1	K14	K5	K100	K1	K1	K1	K1	K1
*fim*H (clade)	22 (B)	30 (C)	30 (C)	41 (A)	22 (B)	22 (B)	22 (B)	22 (B)	22 (B)
*ibe*A (invasion gene)	+	−	−	−	+	+	+	+	+
Virulence-associated plasmid	pS88-like	−	pRS218-like: *sen*B-*cjr*ABC*+*	pRS218-like: *sen*B-*cjr*ABC*+*	pS88-like	pS88-like	pS88-like	pS88-like	pS88-like

### Genomic diversity and virulence architectures of ST131 isolates causing neonatal meningitis

Whole-genome sequencing analysis revealed three *fim*H-defined sublineages: *fim*H22 (*n*=6), *fim*H30 (*n*=2) and *fim*H41 (*n*=1). All isolates carried group 2 capsule loci (*kps*M II), with capsule serotypes including K1 (*n*=6), K5 (*n*=1), K14 (*n*=1) and K100 (*n*=1). The K1 capsular antigen was phenotypically confirmed by agglutination using the Pastorex Meningitis kit (Bio-Rad, France). All six K1 isolates belonged to the O25b:H4 serotype and carried the *fim*H22 allele, consistent with clade B assignment [10].

Beyond the K1 capsule, ST131-O25b-*fim*H22 isolates (clade B) displayed a relatively homogeneous virulence-associated genomic profile enriched in NMEC-associated determinants. Markers of a pS88-like (ColV-type) virulence plasmid and *ibe*A were detected in 6/6 isolates (Table S1, available in the online supplementary material).

Isolates outside the clade B (ST131-O25b-*fim*H22) group displayed more heterogeneous virulence architectures, and none carried the K1 capsule. Two isolates (S246 and S261), belonging respectively to clade C (*fim*H30) and A (*fim*H41), carried a pRS218-like virulence plasmid. The remaining isolate S242 of clade C (*fim*H30) lacked both pRS218-like and pS88-like plasmid signatures, notably *sen*B and *cjr*ABC, but retained several virulence-associated determinants, including the iron acquisition systems *sit*ABCD and *irp*2*–fyu*A as well as the serum resistance gene *iss* [20] (Table S1).

### Antimicrobial resistance profiles of ST131 meningitis isolates

β-lactam resistance was low overall (Table S2): six isolates carried *bla*_TEM-1B_, and S242 was the only ESBL-producing isolate, harbouring *bla*_CTX-M-15_. No carbapenemase genes were identified. Quinolone resistance showed a marked sublineage-specific pattern. ST131-O25b-*fim*H22 isolates remained largely fluoroquinolone-susceptible, with only two exceptions: S424, carrying a *gyr*A mutation, and S398, harbouring a *qnr*S1 gene. Fluoroquinolone resistance was otherwise restricted to non-ST131-O25b-*fim*H22 isolates with mutations in *gyr*A, *par*E+/-*par*C (S242, S246 and S261). More generally, acquired resistance determinants were enriched in the non-ST131-O25b-*fim*H22 group, which displayed higher resistance gene burdens (mean, 9.0; range, 7–10), whereas ST131-O25b-*fim*H22 meningitis isolates carried comparatively few acquired resistance genes (mean, 2.7; range, 0–6) (Tables S2 and 3).

### Phylogenomic placement of neonatal meningitis-associated ST131-O25b-*fim*H22 isolates

To place these ST131-O25b-*fim*H22 isolates within the broader diversity of ST131 clade B, we reconstructed a global phylogenomic dataset using publicly available genomes. This collection included isolates of human origin from urinary tract infections (*n*=163), bacteraemia (*n*=148), previously reported neonatal meningitis cases (*n*=2) [3] and animal-source isolates (*n*=25) [18] for a total of 344 isolates (Table S3, Fig. 1).

**Fig. 1.**
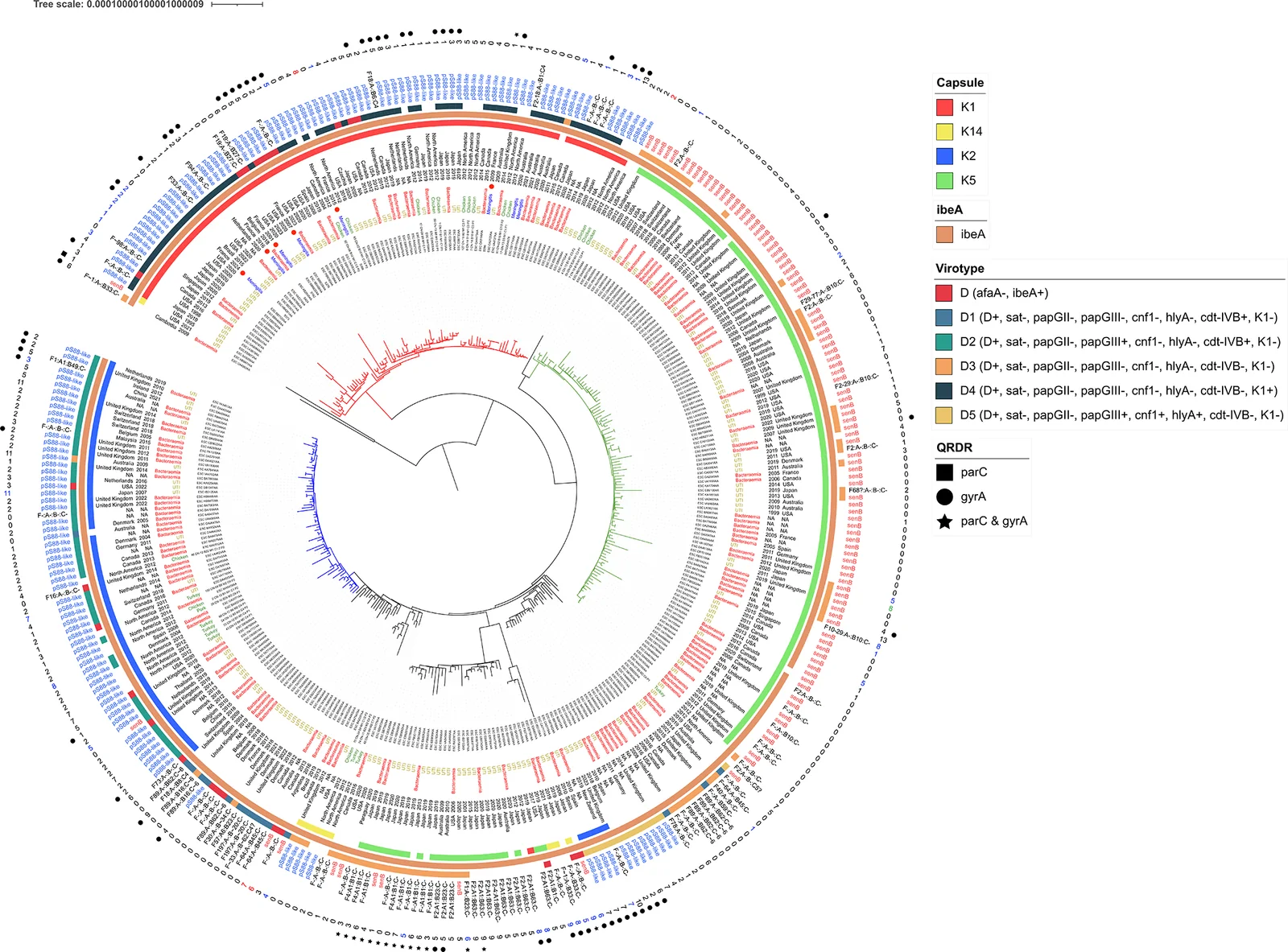
Core-genome phylogeny of ST131-O25b-*fim*H22 isolates, highlighting neonatal meningitis strains within the global ST131-O25b-*fim*H22 lineage. The phylogenetic tree included 344 ST131-O25b-*fim*H22 isolates, comprising neonatal meningitis-associated strains (*n*=8; six French NMEC from this study and two NMEC isolates reported by Nhu *et al*. [3]), urinary tract infections (*n*=163), bacteraemia (*n*=148) and animal-source ST131-H22 isolates derived from the foodborne dataset of Liu *et al*. [18] (*n*=25). Isolates were compiled from our dataset, EnteroBase and published studies (Table S3). From the centre to the periphery, the annotation layers display the strain name, clinical origin, sampling year and country or region of isolation sequentially. Red dots next to the clinical origin indicate French NMEC isolates. The following ring displays the capsular type (K1, K5, K14 or K100), followed by the presence or absence of *ibe*A. The subsequent layer represents the ST131 virotype according to the Blanco *et al*. [10] classification, with virotype D further subdivided into subtypes D1–D5 following the scheme proposed by Mora *et al*. [19], based on their virulence gene profiles. The subsequent ring indicates the presence of pS88-like, pRS218-like (*sen*B-*cjr*ABC-positive) virulence plasmid signatures or other IncF plasmid types. The antimicrobial resistance gene score (0–13) is then indicated, with colour marking the detection of major β-lactamases (blue for ESBLs, red for carbapenemases and green for isolates carrying both). The outermost layer denotes quinolone resistance-determining region (QRDR) mutations using distinct shapes: circles for *gyr*A mutations, squares for *par*C mutations and stars when both loci are mutated.

In the maximum-likelihood phylogeny, French neonatal meningitis-associated ST131-O25b-*fim*H22 isolates (*n*=6) clustered within a well-defined monophyletic cluster of 85 genomes, spanning nearly 3 decades (1995–2024), including the 2 additional meningitis isolates, 25 bacteraemia isolates, 39 urinary tract infection isolates and 13 avian-source isolates. This cluster uniformly fulfilled the criteria for virotype D according to the ST131 virotyping scheme proposed by Blanco *et al*. [10], based on the presence of *ibe*A (85/85). Most isolates also carried the K1 capsule (83/85), a pS88-like virulence plasmid (74/85) and multiple siderophore systems, including aerobactin and yersiniabactin (Table S3, Fig. 1). Using the subtyping scheme of Mora *et al*. [19], in which virotype D4 denotes *ibe*A-positive isolates lacking *sat* and *afa*/*dra*BC, whereas virotype D3 includes isolates carrying one or both of these determinants, the cluster was predominantly assigned to D4 (73/85 isolates). Only two isolates belonged to virotype D3, while ten could not be further subclassified because they lacked the discriminant markers required for this scheme (Fig. 1).

Within the 85-genome neuroinvasive cluster, 78 isolates (91.8%) shared the same HC100 assignment (HC100 : 1709) and 53 (62.4%) remained grouped within the same HC50 cluster (HC50 : 3825), consistent with the long-term persistence of a closely related K1/*ibe*A-positive ST131-O25b-*fim*H22 lineage across human and avian reservoirs (Table S3). To further investigate the distribution of this lineage in non-human reservoirs, we analysed an extended collection of animals- or food-associated ST131-O25b-*fim*H22 genomes retrieved from EnteroBase (*n*=623) (Table S4). Among these isolates, K1 capsule-associated loci were detected in 345/623 isolates (55.4%), and *ibe*A was present in 342/345 of these K1+ isolates (99.1%).

Markers of a pS88-like virulence plasmid signature were identified in 475/623 isolates (76.2%), while pRS218-like markers were detected in 34/623 isolates (5.5%). Of note, 23/34 (67.6%) of the pRS218-like-positive isolates originated from canine or feline sources, and none carried the K1 capsule, contrasting with the K1/*ibe*A-positive profile associated with the pS88-like lineage identified in neonatal meningitis. According to the BlancoMora classification [10, 19], 613/623 isolates (98.4%) belonged to virotype D. Notably, all K1/i*be*A-positive animal isolates (342/342) fulfilled the criteria for virotype D, and the majority belonged to the D4 subtype (303/342, 88.6%), mirroring the virotype composition observed within the neuroinvasive cluster.

Comparison with the HierCC profiles of the neuroinvasive cluster revealed that 394 animal- and food-associated isolates belonged to the same HC100 lineage, including 266 isolates sharing HC50 (Table S4). Among these closely related animal- and food-associated isolates, 248/266 (93.2%) carried the combined K1/*ibe*A-positive profile, and 243/248 (98.0%) also harboured a pS88-like virulence plasmid signature. Poultry-associated isolates represented the majority of these closely related genomes (222/243, 91.3%), suggesting the existence of a widespread avian-associated reservoir closely related to the neuroinvasive ST131-O25b-*fim*H22 lineage identified in humans.

## Discussion

In this 25-year nationwide study including 355 NMEC isolates, 9 ST131 isolates (2.5%) were identified, indicating that although uncommon, ST131 remains a persistent cause of NMEC. This contrasts with the global predominance of ST131 in urinary tract infections and bacteraemia, largely driven by expansion of the H30 sublineage [9, 21]. Despite its limited representation, ST131 meningitis isolates displayed a strikingly non-random population structure, with most belonging to the clade B/ST131-O25b-*fim*H22 lineage (*n*=6). These isolates retained a pathogenicity-associated genetic profile classically associated with neonatal meningitis, including the K1 capsule, *ibe*A (virotype D) and a pS88-like ColV plasmid. The clade B/ST131-O25b-*fim*H22 lineage predates the emergence of the globally dominant H30 lineage, which appears comparatively less enriched in virulence determinants associated with neuroinvasion [3, 11, 13, 21, 22].

Interestingly, among the three non-ST131-O25b-*fim*H22 isolates, two lacked the K1 capsule (S246, *fim*H30 and S261, *fim*H41) but carried a pRS218-like virulence plasmid, classically associated with ST95 NMEC strains [15, 16]. This plasmid encodes multiple virulence determinants shown in brain microvascular endothelial cell (hBMEC) models to enhance endothelial invasion and bloodstream persistence [15]. The remaining isolate (S242) lacked both the K1 capsule and typical virulence plasmids (pS88-like or pRS218-like) associated markers *sen*B and *cjr*ABC, while retaining several other virulence modules (Table S1). This isolate was also the only ESBL producer, harbouring CTX-M-15, consistent with our previous report of fatal ST131 NMEC infections associated with ESBL production [20].

These findings support the idea that non-ST131-O25b-*fim*H22, including clade C lineages, may cause neonatal meningitis through plasmid-mediated virulence mechanisms, even in the absence of K1 capsule, as previously reported in rare NMEC cases [23, 24]. Such infections are also likely facilitated by host-related factors, particularly prematurity, blood–brain barrier immaturity and impaired neonatal immune defences.

Our phylogenomic analysis showed that neonatal ST131-O25b-*fim*H22 isolates clustered within a monophyletic group of 85 genomes comprising meningitis, bacteraemia, UTI and avian-associated isolates from diverse geographic origins (Table S3). All but two isolates carried the K1 capsule, and most harboured pS88-like virulence plasmids, indicating a highly conserved pathogenicity-associated genomic backbone. The majority of isolates belonged to virotype D4 (*n*=73), further supporting the existence of a structured and genetically coherent ST131-O25b-*fim*H22 sublineage enriched in neuroinvasive-associated traits [19].

The HierCC analysis further reinforces the evolutionary stability of this lineage. Although the neuroinvasive cluster encompassed isolates recovered over nearly three decades from multiple continents and diverse ecological sources, the vast majority remained grouped within the same HC100 lineage, with more than half further clustering at the HC50 level. Such limited diversification despite broad temporal and ecological distribution suggests the long-term persistence of a successful ancestral K1/*ibe*A-positive ST131-O25b-*fim*H22 cluster.

Our extended analysis of 623 animal-associated ST131-O25b-*fim*H22 genomes further supports this interpretation. More than half carried the K1 capsule, almost all K1-positive isolates also harboured *ibe*A, and over three-quarters displayed a pS88-like virulence plasmid signature. This pattern was highly concordant with virotype assignment: all K1/*ibe*A-positive animal isolates fulfilled the criteria for virotype D, most belonging to subtype D4, thereby closely mirroring the pathogenicity-associated genomic architecture of the human neuroinvasive cluster. HierCC analysis further showed that 394/623 animal-associated isolates belonged to the same HC100 lineage, including 266 that remained grouped at HC50. Within this closely related HC50 subset, nearly all isolates carried the combined K1/*ibeA*/pS88-like profile and were predominantly from poultry-associated sources. These findings extend previous reports of poultry-associated ST131-O25b-*fim*H22 isolates and support the existence of a broad avian-associated reservoir closely related to human neuroinvasive isolates [18, 25]. Notably, the predominance of virotype D/D4 is consistent with experimental studies showing that ST131 virotypes differ in virulence in murine infection models, with virotype D isolates exhibiting increased pathogenicity. This functional evidence further supports the high pathogenic potential of the closely related human- and poultry-associated D4 lineage [19].

Experimental evidence further supports the close relationship between APEC and NMEC lineages. Tivendale *et al*. demonstrated that selected poultry-associated O18 APEC strains shared marked genomic and virulence similarity with human NMEC lineages, including closely related large ColV-like virulence plasmids carrying conserved ExPEC-associated determinants (*iro*N*, iuc, iss, hly*F and *omp*T), and were capable of inducing bacteraemia and meningitis in the neonatal rat model [14]. Peigne *et al*. showed that the pS88 virulence plasmid, a major determinant of NMEC pathogenicity, is closely related to plasmids found in APEC and circulates across meningitis-associated, avian and human ExPEC lineages [13].

Notably, the *sen*B-*cjr*ABC-positive pRS218-like signature showed a distinct ecological distribution from the K1/*ibe*A/pS88-like background. In the extended animal-associated dataset, this marker combination was uncommon, mainly detected in canine and feline isolates, and absent from K1-positive backgrounds (Table S4). This pattern is consistent with previous reports of ST131 and other ExPEC lineages shared between humans and household pets, although broader genomic surveys suggest that dogs and cats are more likely to represent spillover hosts than primary reservoirs of these lineages [26–28].

These contrasting ecological distributions suggest that distinct virulence plasmids may be associated with different evolutionary trajectories within ST131 and may persist through a broader One Health cycle. Such ecological persistence may be facilitated by the selective advantages conferred by the K1 capsule across diverse host and environmental niches. Recent evolutionary studies have shown that the K1 capsule has been acquired independently multiple times across diverse ExPEC lineages and is strongly maintained by selection because it enhances serum survival and systemic persistence [29]. Beyond invasive disease, K1 expression has also been associated with resistance to innate environmental predators such as free-living amoebae, which are increasingly recognized as ecological drivers shaping ExPEC virulence evolution [30].

In this evolutionary context, our findings suggest that ST131-O25b-*fim*H22 represents a specialized K1-positive sublineage that has preserved a stable and highly conserved pathogenicity-associated genomic architecture. In contrast to the globally dominant *fim*H30 lineage, whose expansion was largely driven by antimicrobial resistance and adaptation to antibiotic-rich clinical environments, ST131-O25b-*fim*H22 isolates remained mostly antibiotic-susceptible, consistent with an ancestral pre-H30 background [9, 21]. This persistence despite the global success of H30 indicates that distinct ST131 sublineages occupy partially divergent ecological and evolutionary niches. While H30 achieved pandemic spread through resistance-driven expansion, ST131-O25b-*fim*H22 may have been maintained by a virulence- and colonization-oriented fitness strategy supported by K1-mediated immune evasion and circulation across animal and foodborne reservoirs. Together, these observations suggest divergent evolutionary trajectories within ST131, balancing resistance specialization with virulence-associated persistence [31].

Nevertheless, the identification of ESBL- or carbapenemase-producing isolates within global ST131-O25b-*fim*H22 populations indicates that this ancestral virulent lineage also has the capacity to acquire clinically important resistance determinants (Table S2). These observations underscore the importance of integrated genomic surveillance across human, animal and food reservoirs to better anticipate the persistence, dissemination and potential resistance evolution of high-risk ExPEC lineages.

The main limitation of this study is the small number of ST131 NMEC isolates, which should be interpreted in light of the rarity of neonatal *E. coli* meningitis and the low frequency of ST131 among NMEC. Additional limitations include potential sampling bias in public genome collections, short-read marker-based plasmid inference and the absence of functional or direct transmission evidence. Nevertheless, by combining a 25-year nationwide NMEC collection with global phylogenomics, this study identifies a coherent K1/*ibe*A-positive ST131-O25b-*fim*H22 lineage enriched in pS88-like signatures and closely related to poultry-associated genomes. This ancestral, virulence-specialized lineage appears to persist across human- and avian-associated sectors, outside the resistance-driven H30 narrative, highlighting the need for integrated One Health genomic surveillance of high-risk ExPEC lineages.

## Supplementary material

10.1099/mgen.0.001857 Supplementary Material 1.

10.1099/mgen.0.001857 Supplementary Material 2.

10.1099/mgen.0.001857 Supplementary Material 3.

## Abbreviations

APEC: avian pathogenic *E. coli*
CSF: cerebrospinal fluid
ESBL: extended-spectrum β-lactamase
ExPEC: extraintestinal pathogenic *E. coli*
hBMEC: human brain microvascular endothelial cell
*ibe*A: *invasion of brain endothelium* A
NMEC: neonatal meningitis *E. coli*
NRC: National Reference Center
STc: Sequence Type complex
