# Supplementary material for: Escherichia coli ST131 isolates are rare in human meningitis but belong to a distinct O25b-fimH22 clade B lineage shared with avian pathogenic Escherichia coli
Source: Microb Genom. 2026 Oct 7;12(10):001857. doi: 10.1099/mgen.0.001857 (PMC13689937; doi:10.1099/mgen.0.001857)
Supplement: Supplementary file 1 [file mgen-12-01857-s001.pdf]

**Strains**	**S162**	**S242**	**S246**	**S261**	**S276**	**S296**	**S398**	**S424**	**S573**
*fim*H (clade)	22 (B)	30 (C)	30 (C)	41 (A)	22 (B)	22 (B)	22 (B)	22 (B)	22 (B)
*ibe*A	+	-	-	-	+	+	+	+	+
Virulence associated plasmid	pS88-like	-	pRS218: *sen*B+ *cjr*ABC+	pRS218: *sen*B+ *cjr*ABC+	pS88-like	pS88-like	pS88-like	pS88-like	pS88-like
*sit*ABCD	+	+	+	+	+	+	+	+	+
*asl*A	+	+	+	+	+	+	+	+	+
*fde*C	+	+	+	+	+	+	+	+	+
*ecp*ABCDER	+	+	+	+	+	+	+	+	+
tsh	+	-	-	-	-	+	+	-	-
*hly*F	+	-	-	-	+	+	+	+	+
*omp*Tp	+	-	-	-	+	+	+	+	+
ets	+	-	-	-	+	+	+	-	+
iss	+	+	+	-	+	+	+	+	+
*iro*BCDEN	+	-	-	-	+	+	+	+	+
*mch*F	+	-	-	-	+	+	-	+	+
*mch*BC	+	-	-	-	-	-	-	-	-
cva, cvi	+	-	-	-	+	+	-	+	+
*iuc*ABCD, *iut*A	+	+	+	+	+	+	+	-	+
*chu*ASTUVWXY	+	+	+	+	+	+	+	+	+
*omp*A	+	+	+	+	+	+	+	+	+
*irp*2, *fyu*A	+	+	+	+	+	+	+	+	+
iha	+	+	+	+	-	-	+	-	-
*kps*DM	+	+	+	+	+	+	+	+	+
*neu*C, *kps*T	+	-	-	-	+	+	+	+	+
*pap*IBX	-	+	+	+	-	-	-	-	-
sat	-	+	+	+	-	-	-	-	-
*sen*B	-	-	+	+	-	-	-	-	-
*cjr*ABC	-	-	+	+	-	-	-	-	-
cea	-	-	-	-	-	+	-	-	-
usp	+	+	+	+	+	+	+	+	+
ast	-	-	-	-	+	-	-	-	-
hek	-	-	-	-	-	-	-	+	+
hra	-	+	-	-	-	-	-	-	-
*ire*A	-	-	-	-	-	-	-	+	+
*ibe*A	+	-	-	-	+	+	+	+	+
cba	+	-	-	-	-	-	-	-	-
*ter*C	-	-	-	-	+	-	-	-	-
cma	+	-	-	-	-	-	+	+	-
*fae*CDEFIJ	+	-	-	-	-	-	-	-	-

Supplementary Table S1 Virulence-associated features of *E. coli* ST131 NMEC isolates

Strains	*S162*	*S242*	*S246*	*S261*	*S276*	*S296*	*S398*	*S424*	*S573*
*fim*H (clade)	22 (B)	30 (C)	30 (C)	41 (A)	22 (B)	22 (B)	22 (B)	22 (B)	22 (B)
blaTEM-1B (penicillinase)	-	+	+	+	+	-	+	+	-
bla_{CTX-M}		$bla_{CTX-M-15}$							
*qnr*S1(quinolone resistance gene)	-	-	-	-	-	-	+	-	-
Mutations on QRDR	-	*gyr*A p.S83L,p.D87N *par*E p.I529L *par*C p.S80Ip.E84V	*gyr*A p.S83L,p.D87N *par*E p.I529L *par*C p.S80Ip.E84V	*gyr*A p.S83L *par*E p.I529L	-	-	-	*gyr*A p.S83L	-
Aminoglycoside resistance genes	-	*aad*A5	*aac*(3)-IId, *aad*A5, *aph*(3")-Ib, *aph*(6)-Id	*aac*(3)-IId, *aad*A5, *aph*(3")-Ib, *aph*(6)-	*aph*(3")-Ib, *aph*(6)-Id	-	*aad*A1 (n=1)	-	-
*mph*A (macrolide resistance gene)	-	+	+	+	-	-	-	-	-
tet (tetracycline resistance genes)	*tet*A	*tet*A	*tet*A	*tet*A	*tet*B	-	*tet*A	-	-
sul (sulfamethoxazole resistance genes)	-	*sul*1	*sul1*, *sul*2	*sul*1, *sul*2	*sul*2	-	*sul*1	*sul*2	-
*dfr*A (trimethoprim resistance genes)	-	*dfr*A17	*dfr*A17	*dfr*A17	*dfr*A5	-	*dfr*A1	*dfr*A1	-

Supplementary Table S2 Antimicrobial resistance determinants of *E. coli* ST131 NMEC isolates

Supplementary Table S3 Characteristics of 344 *E. coli* ST131-O25b-*fim*H22 isolates included in the global phylogenetic analysis: The table includes six french neonatal meningitis-associated ST131-O25b-*fim*H22 isolates from this study, two previously published neonatal meningitis isolates from Nhu *et al.(3)*, food/animal-source ST131-O25b-*fim*H22 isolates from the Liu *et al.* dataset (18) (n = 25) and additional ST131-O25b-*fim*H22 isolates retrieved from EnteroBase (n = 311) encompassing bacteremia (n = 148) and urinary tract infection (n = 163).

Supplementary Table S4. Characteristics of the extended collection of 623 animal- and food-associated and foodborne *Escherichia coli* ST131-O25b-*fim*H22 isolates retrieved from EnteroBase (1990-2026), including HierCC assignments and virulence-associated features.
